# Antithrombotic therapy and outcomes of cervical arterial dissection in the trauma patient: a case series

**DOI:** 10.1186/1752-2897-4-13

**Published:** 2010-12-13

**Authors:** Holly E Hinson, M JB Stallmeyer, Jon P Furuno, Karen L Yarbrough, John W Cole

**Affiliations:** 1 Department of Neurology, University of Maryland School of Medicine, Baltimore, MD, USA; 2 Department of Diagnostic Radiology and Nuclear Medicine, University of Maryland School of Medicine, Baltimore, MD, USA; 3 Department of Epidemiology and Public Health, University of Maryland School of Medicine, Baltimore, MD, USA; 4 Veterans Affairs Maryland Health Care System, Baltimore, MD, USA

## Abstract

**Background:**

The use of antithrombotic therapy (anticoagulants and/or antiplatelets) in the setting of traumatic cervical arterial dissection (CAD) for the prevention of stroke remains controversial. This issue is further complicated by the frequent co-existence of intracranial hemorrhage (ICH) and other intracranial injuries, and also the wide variability in treatment due to a lack of evidence-based guidance. To address these controversies, a registry in a major Level I trauma center was created. The purpose of this investigation was to compare the safety of antithrombotic therapy in post-traumatic CAD. Analysis from the first year is presented.

**Methods:**

All cervical dissections from the year 2005 were identified in patients at least 18 years of age by diagnosis code from radiology and trauma databases. Presence of arterial injury and grade, and other intracranial disease or injury such as stroke was diagnosed by a trauma radiologist and adjudicated by a neuroradiologist.

**Results:**

Fifty-five patients with cervical artery dissection were identified. Fourteen patients presented with a total of 20 acute, post-traumatic intracranial hemorrhages (ICH). Seven of the 14 patients with ICH were treated with antithrombotic therapy, and none extended their intracranial hemorrhages. Of the 41 patients without pre-existing ICH, 28 were treated with antithrombotic therapy and only one developed an interval hematoma. Among all 55 cases, two patients developed an acute ischemic stroke in the territory of the dissected artery after admission; both patients were in the untreated group.

**Conclusion:**

In so far as antithrombotic therapy may offer benefit in preventing early ischemic stroke following cervical artery dissection, these data suggest withholding antiplatelet or other antithrombotics following trauma may not be warranted, even in the setting of intracranial hemorrhage. From a safety perspective, this registry-based case series indicates antithrombotic management of arterial injury did not contribute to development or progression of ICH, even in patients with pre-existing ICH. This data suggest that instituting early antithrombotic therapy presents a low risk of ICH or hemorrhage extension among traumatic cervical dissection patients.

## Background

Cervical arterial injuries can occur after blunt trauma, and are often seen in the setting of **w**hiplash injuries resulting from forced neck flexion and extension. Such injuries are a well-recognized risk factor for ischemic stroke secondary to either artery to artery embolic phenomena, or from hemodynamic compromise due to luminal stenosis or occlusion [1,2]. Dissection accounts for approximately 2% of all ischemic stroke, but perhaps as much as 20% of ischemic stroke in patients less than 45 years of age [3,4]. Although the incidence of all traumatic cervical arterial injuries is unknown, the estimated incidence of carotid dissection is 0.08% of all blunt traumas [5] and 0.67% of MVA-related blunt trauma [6]. The combined incidence of carotid and vertebral injury, also known as blunt cerebrovascular injury (BCVI), is estimated at 1% of all blunt traumas [7]. With the increased use of multi-detector computer tomography, identification of these injuries is becoming more common.

Much of the current literature on the natural history of dissection and stroke focuses on therapy for "spontaneous dissection", rather than traumatic dissection [8-11]. Ischemic stroke, primarily from artery to artery emboli after arterial injury, can occur at any time after injury, but is most frequent in the first 7 days [12,13]. The timing and use of antithrombotic therapy, including both anticoagulants and antiplatelets, for stroke prevention in dissection remains controversial due to the absence of prospective randomized clinical trials. The appropriate use of antithrombotic therapy is even less clear in trauma patients, who frequently have other serious injuries associated with increased hemorrhage risk. Our group initiated a registry for the methodologies of identification, treatment, and outcome of cervical dissection at a Level I trauma center. Data from the first year is presented.

## Methods

### Study Design and Case Identification

The R. Adams Cowley Shock Trauma Center is a Level I trauma center in Baltimore, MD, which receives approximately 8,265 patient visits annually. Trauma patients are routinely screened within 6 hours of admission for vascular injury utilizing an intravenous contrast-enhanced whole-body multi-detector computed tomography scanning protocol. All dissections in patients at least 18 years of age during 2005 were identified by diagnosis code (either carotid or vertebral dissection) from Radiology and Trauma databases populated from the multi-detector CT results. A consecutive, case series was collected in a retrospective manner from chart review. Presence of arterial injury, ischemic or hemorrhagic stroke, and other intracranial disease or injury was diagnosed by a trauma radiologist and adjudicated by a neuroradiologist. This protocol was approved by University of Maryland, Baltimore Institutional Review Board prior to study commencement.

### Data Collection and Outcomes Measures

Charts of identified subjects were reviewed with emphasis on discharge summaries, physical therapy notes and follow-up clinic visits. All data were abstracted using a standardized data collection form. Information on demographics and mechanism of injury were obtained from admission notes. Locations and grade of injury were obtained from radiology reports and verified by a neuroradiologist. Information regarding antithrombotic therapy treatment and type, as well as hospital course and interim complications, were noted from discharge summaries. Outcomes were quantified by calculating a Glasgow Outcome Score (GOS) ranging from 2 (persistent vegetative state) to 5 (good recovery) as assessed at a follow up clinic appointment closest to twelve weeks from the injury. Cases were assigned a 5 if the subject could resume normal occupational and social activities, 4 if the subject was independent but unable to resume prior activities, 3 if partially or totally dependent on other for activities of daily living, and 2 if the subject was unaware of surroundings or unable to respond to stimuli [14].

All data were entered in a database (MS Excel, Redmond, WA) and data were analyzed using SAS statistical software, version 9.1 (SAS Institute, Cary, NC).

## Results

In total, 55 patients with dissection were identified. Characteristics of the study population in total and stratified by whether the patient received antithrombotic therapy are displayed in Table 1. Mean age was approximately 47 years and approximately 58% of the patients were male. Among the 55 patients, there were 68 arterial dissections of which 22 were vertebral, 44 were of the internal carotid, and 2 were of the common carotid. Most patients' injuries were the result of a motor vehicle crash and prevalence of hemorrhage on presentation was approximately 26%. Fourteen patients presented with a total of 20 post-traumatic hemorrhagic injuries including subdural hematoma (n = 6), subarachnoid hemorrhage (n = 9), intraparenchymal hemorrhage (n = 2), and intraventricular hemorrhage (n = 3). Six of the 14 patients had multiple types of hemorrhage.

**Table 1:** Characteristics of the Study Population

	Treatment (n = 35)	No Treatment (n = 20)	All (n = 55)
Age, mean (SD)	47.7 (18.7)	46.1 (19.1)	47. 1(18.7)
Male Sex, n (%)	18 (51.4)	14 (70.0)	32 (58.2)
Location of Arterial Injury†			
Common Carotid	2	0	2
Internal Carotid	29	15	44
Vertebral	14	8	22
Mechanism of Injury, n (%)			
Motor Vehicle Crash	30 (85.7)	15 (75.0)	45 (81.8)
Fall	4 (11.4)	4 (20.0)	8 (14.6)
Other	1 (2.9)	1 (5.0)	2 (3.6)
Hemorrhage on Presentation*	7 (20.0)	7 (35.0)	14 (25.5)
GCS on Presentation, median (IQR)	15 (6-15)	13.5 (3.5-15.0)	15 (6-15)

SD: standard deviation; GCS: Glasgow Coma Score; IQR: interquartile range

†Among the 55 patients, there were 68 arterial injuries and 1 aneurysm.

* 14 patients presented with a total of 20 intracranial hemorrhages (ICH), specifically subdural hematoma (n = 6), subarachnoid hemorrhage (n = 9), intraparenchymal hemorrhage (n = 2), or intraventricular hemorrhage (n = 3).

Table 2 displays the distribution of treatment types stratified by whether the patient had a hemorrhage on initial CT scan. Seven of the 14 patients with a hemorrhage were treated with antithrombotic therapy (6 with antiplatelets, and 1 with a combination of antiplatelets and anticoagulation). Of the 41 patients without pre-existing hemorrhage, 28 were treated with antithrombotic therapy (22 with antiplatelets alone, 3 with anticoagulation, and 3 with a combination of antiplatelets and anticoagulation). None of the treated patients with hemorrhage on initial presentation either extended their hemorrhage or developed ischemic stroke. Of the 28 treated patients who did not a have a hemorrhage on presentation, only one developed interval ICH. This subject presented with a left common carotid dissection and was placed on aspirin, but subsequently suffered a cardiac arrest from hemodynamic instability due to thoracic injuries. He then developed extensive left hemispheric ICH and subsequently expired. In summary, no treated patient developed an ischemic stroke.

**Table 2:** Type of treatment by presence or absence of hemorrhage on presentation (n = 55)

Hemorrhage on presentation (n)	Anti-platelet only n (%)	Anti-coagulant only n (%)	Combination therapy n (%)	No treatment n (%)
Yes (14)	6 (42.9)	0	1 (7.1)	7 (50.0)
No (41)	22 (53.7)	3 (7.3)	3 (7.3)	13 (31.7)

There were 20 patients that were not treated, 7 with pre-existing ICH and 13 without pre-existing ICH. In total, two untreated patients experienced ischemic strokes in territories ipsilateral to the vascular injury. None of the untreated group extended their hemorrhages. Patients were not treated with antithrombotic agents to prevent ischemic stroke for a variety of reasons, most commonly due to rapid expiration shortly after presentation or due to need for emergent surgical procedures.

In total, eight patients died in hospital, 7 of those in the non-treatment group (n = 20) and 6 of these within the first 7 days of hospitalization. The subject who expired in the treatment group presented with both an ICH and SAH, and was treated with clopidogrel for a left ICA dissection. While the patient experienced no rebleeding as associated with therapy, a poor prognosis led the patient's family to electively withdraw care. Among the patients that were not treated, 7 with pre-existing ICH and 13 without pre-existing ICH, two patients with pre-existing ICH died. One patient expired secondary to cardiac arrest and the other patient from brain herniation related to cerebral edema secondary to anterior and middle cerebral artery infarction ipsilateral to an internal carotid artery dissection.

Among patients alive at hospital discharge (n = 47), median (IQR) modified Rankin scores were 4 (1-5) and 2 (0-3) in the treated and untreated groups respectively (Table 3). Among the 47 patients discharged alive, 39 patients returned to outpatient clinic for evaluation and 8 patients were lost to outpatient follow up. Median Glasgow Outcome Scores at three months were 4 (3-5) and 4 (4-5) in the treated and untreated groups respectively (Table 3).

**Table 3:** Outcomes of patients with traumatic cervical arterial dissection as stratified by antithrombotic treatment and the absence/presence of hemorrhage at presentation.

	Treatment		No Treatment	
	
	n = 35		n = 20	
	n	median (IQR)	n	median (IQR)
Modified Rankin Score at Discharge (n = 47)*	34	4 (1-5)	13	2 (0-3)
Hemorrhage	6	4.5 (4-5)	3	0 (0-5)
No Hemorrhage	28	2.5 (0-3)	10	2 (1-5)
Glasgow Outcome Score at 3 Months (n = 39)†	31	4 (3-5)	8	4 (4-5)
Hemorrhage	8	3 (3-4)	3	5 (4-5)
No Hemorrhage	23	4 (3-5)	5	4 (4-4.5)

IQR: interquartile range

*8 patients missing due to death during the index admission

†16 patients missing: 8 died on index admission and 8 did not return for scheduled 3 month follow-up visit.

## Discussion

We observed no hemorrhagic complications related to stroke prevention with antithrombotic therapy in this case series of cervical arterial dissection patients. Our results are of interest in the trauma population, as there is understandable reluctance for early treatment of these injuries with anti-platelets or anticoagulation due to concerns for promoting hemorrhage. Previous small, retrospective studies have suggested both anti-platelets and anticoagulants are safe for use in arterial dissection [2], but no distinction was made between traumatic dissection and spontaneous dissection in those trials. Regarding trauma patients, a case series by Fabian et al. evaluating blunt cerebrovascular injury demonstrated 2 of 47 patients receiving heparin developed subdural hematomas requiring evacuation and one patient had worsening intraventricular hemorrhage requiring decreased heparin dose. All three made uneventful recoveries after these complications [6]. Cothren et al. reported only 2 patients developing complications from treatment in their series of 73 blunt carotid injuries receiving antithrombotic therapy, namely visceral bleeding in both cases. The authors suggest more aggressive anti-coagulation protocols might be used based on this observation, although cases of intracranial hemorrhage were excluded from the treatment group [15].

Our case series is unique in than it represents one of the larger case series of traumatic cervical dissection in the literature, and includes patients with intracranial hemorrhage in the treatment group. Additionally, given that the majority of ischemic strokes occur early in the course of arterial dissection, most often in the first week, our method of case review and follow-up would have identified such events. As such, we are confident that no ischemic or hemorrhagic strokes went unidentified throughout the hospitalization and follow-up period.

A major limitation of our study was the lack of standardized protocols regarding the timing, dosing, and selection of the antithrombotic agents implemented. Patient selection and the decision to use (or not use) antithrombotic therapy was performed at the discretion of the treating physician, and often a rationale was not noted. The focus of this study was intracranial complications as related to dissections or from antithrombotic therapy, as such, subjects may have experienced hemorrhage in other locations, such as GI bleeding, that was not captured in this analysis. However all in hospital deaths were identified. Another limitation of the study is the small number of study subjects, particularly among those that received only anticoagulation and/or combination therapy, as we only identified seven such individuals. Given this limitation we urge the reader caution when interpreting these specific results. Nonetheless we felt it was important to include this information for completeness and to demonstrate that such therapies are implemented in practice. Lastly, our study did not consider the effects of other potential conditions in this cohort that might affect hemorrhagic risk; these might include the size of initial hemorrhage, coagulopathies from infection, metabolic disturbances, end-organ damage, perisurgical risks, or the use of LMWH for DVT prophylaxis. Future work should include protocols for patient inclusion and exclusion from antithrombotic therapy as well as standardized medication timing and dosing. All hemorrhagic complications, both CNS and otherwise, should be recorded during hospitalization and follow-up.

## Conclusion

In so far as antithrombotic therapy may offer benefit in preventing early ischemic stroke following cervical artery dissection, these data suggest withholding antiplatelet or other antithrombotics following trauma may not be warranted, even in the setting of intracranial hemorrhage. From a safety perspective, this registry-based case series indicates antithrombotic management of arterial injury did not contribute to development or progression of ICH, even in patients with pre-existing ICH. Our data suggests that early therapy might be safe in trauma patients from the perspective of worsening intracranial injury.

## Competing interests

The authors declare that they have no competing interests.

## Authors' contributions

HEH identified the study participants and compiled the database. HEH, MJBS, KLY, and JWC conceived of the study, and participated in its design and coordination and helped to draft the manuscript. JPF and JWC performed the statistical analysis. All authors read and approved the final manuscript.
